# Cardiomyocyte electrophysiology and its modulation: current views and future prospects

**DOI:** 10.1098/rstb.2022.0160

**Published:** 2023-06-19

**Authors:** Christopher L.-H. Huang, Ming Lei

**Affiliations:** ^1^ Physiological Laboratory, University of Cambridge, Downing Street, Cambridge CB2 3EG, UK; ^2^ Department of Biochemistry, University of Cambridge, Tennis Court Road, Cambridge CB2 1QW, UK; ^3^ Department of Pharmacology, University of Oxford, Mansfield Road, Oxford OX1 3QT, UK

**Keywords:** cardiac arrhythmia, cardiac rhythm, ion channels, Ca^2+^ homeostasis, metabolic oxidation, cardiac remodelling

## Abstract

Normal and abnormal cardiac rhythms are of key physiological and clinical interest. This introductory article begins from Sylvio Weidmann's key historic 1950s microelectrode measurements of cardiac electrophysiological activity and Singh & Vaughan Williams's classification of cardiotropic targets. It then proceeds to introduce the insights into cardiomyocyte function and its regulation that subsequently emerged and their therapeutic implications. We recapitulate the resulting view that surface membrane electrophysiological events underlying cardiac excitation and its initiation, conduction and recovery constitute the final common path for the cellular mechanisms that impinge upon this normal or abnormal cardiac electrophysiological activity. We then consider progress in the more recently characterized successive regulatory hierarchies involving Ca^2+^ homeostasis, excitation–contraction coupling and autonomic G-protein signalling and their often reciprocal interactions with the surface membrane events, and their circadian rhythms. Then follow accounts of longer-term upstream modulation processes involving altered channel expression, cardiomyocyte energetics and hypertrophic and fibrotic cardiac remodelling. Consideration of these developments introduces each of the articles in this *Phil. Trans. B* theme issue. The findings contained in these articles translate naturally into recent classifications of cardiac electrophysiological targets and drug actions, thereby encouraging future iterations of experimental cardiac electrophysiological discovery, and testing directed towards clinical management.

This article is part of the theme issue ‘The heartbeat: its molecular basis and physiological mechanisms’.

## 1. Classical experiments: Silvio Weidmann (1921–2005)

The heart is the most important and prominent biological oscillator and is critical to most multicellular animal life. Its functional disruption causes death or disease. Understanding both normal and abnormal cardiomyocyte physiology is thus of fundamental scientific and clinical importance. It involves mechanisms operating at multiple cellular levels, ranging from the cell membranes and their molecular and cellular signalling machinery, through function in entire atrial and ventricular chambers and their conducting and pacing tissue, to systemic modulation by central and peripheral nervous and endocrine mechanisms. Much of this area and its application date from Silvio Weidmann's (1921–2005) pioneering experiments. This article and this *Phil. Trans. R. Soc.* issue it introduces, prefaced by DiFrancesco & Noble [1], falls close to and celebrates Weidmann's 100th birthday.

Weidmann was first to record accurate cardiomyocyte action potentials (APs), the functional basis of cardiac electrophysiological activation, in the 1950s, employing recently invented Ling–Gerard glass microelectrodes [2]. He demonstrated and clarified the contributions of Na^+^ and K^+^ currents, *I*_Na_ and *I*_K_, to the initiation and conduction of excitation and its subsequent repolarization and recovery from refractoriness. Ventricular, atrial and Purkinje cardiomyocyte APs showed relatively rapid (less than 1 ms) upstrokes whose amplitude, in contrast to background resting potentials, depended upon extracellular [NaCl]. This implicated a selective transient Na^+^ permeability [3] reflecting a local anaesthetic-sensitive, *inward* voltage-dependent *I*_Na_ [4,5] paralleling findings in nerve. The subsequent, more gradual, AP recoveries to the resting potential varied in timescale and waveform between atria, and ventricles and Purkinje fibres with their prolonged plateau phases [6]_._ Membrane impedance determinations identified the recoveries with inward rectifying rapid *outward* K^+^ current, *I*_Kr_. Following recovery, Purkinje fibres additionally showed depolarizing *pacemaker currents*, potentially leading to re-excitation and repetitive activity. Weidmann's work then anticipated connexin gap junction-mediated AP propagation [7,8] and relationships between membrane voltage, extracellular Ca^2+^ and contraction [9].

## 2. Cardiac arrhythmias: a major public health problem

These early observations were key to the development of the cardiac electrophysiological field and the continuing productive and constructive dialogue between its fundamental science and clinical applications bearing on normal and abnormal cardiac activity. The latter results in the major public health problem of cardiac arrhythmias, a leading cause of clinical mortality and morbidity, second in incidence only to all cancers combined. Sinus node disorders (SND) form the major indication for pacemaker implantation worldwide. Atrial fibrillation (AF) affects 1 : 10 adults aged >60 years [10–12], increasing stroke incidences and all-cause mortality [12]. Ventricular arrhythmias precipitating sudden cardiac death (SCD) are a major cause of mortality in cardiac failure, and associated metabolic, including common diabetic and ischaemic, conditions [13].

**Figure 1.**
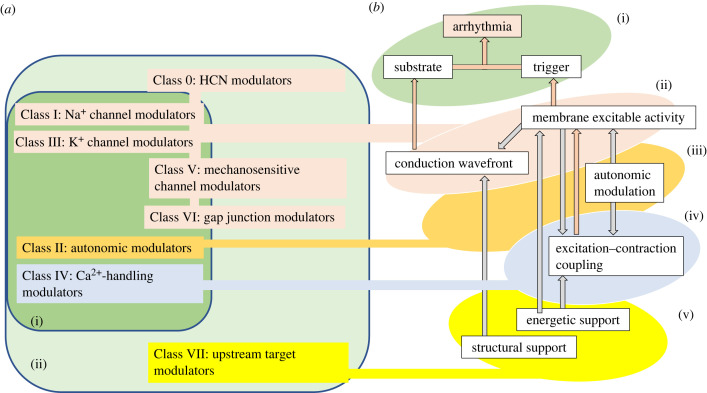
Mapping of physiological targets onto successive levels of cardiomyocyte function. (*a*) Classification of cardiotropic agents as suggested initially by Vaughan Williams (i) and then by subsequent work (ii) demonstrating subsequently discovered Class I–VII targets. These involve a wide range of surface and intracellular membrane ion channels, ion exchangers, transporters, autonomic receptors, ionic pumps, and energetic and structural remodelling processes. Nevertheless, these map onto (*b*) a hierarchy of physiological processes all potentially contributing to (i), triggering and re-entrant arrhythmic events. These levels of cardiomyocyte regulation successively involve (ii) ion channels in the cardiomyocyte membrane and their modification by (iii) autonomic signalling, (iv) excitation–contraction coupling and (v) longer-term upstream energetic or structural remodelling targets (adapted from fig. 1 of [14]). (Online version in colour.)

The early cardiac electrophysiological studies led to the classical Singh–Vaughan Williams classification scheme simultaneously classifying physiological targets governing cardiac rhythm and the then known cardiotropic drugs (figure 1*a*(i)) [15,16]. It provided widely useful clinical guidelines [17]. Here, Class I drugs targeted *I*_Na_, reducing AP phase 0 slopes and overshoots, paralleling Weidmann's findings [18], and varyingly affecting AP duration (APD) and effective refractory period (ERP). Class II β-adrenergic inhibitors slowed sino-atrial node (SAN) pacing and atrioventricular node (AVN) conduction [19,20]. Class III voltage-gated K^+^ channel blockers delayed AP phase 3 repolarization, lengthening ERPs. Class IV L-type Ca^2+^ channel inhibitors reduced cardiac, particularly SAN and AVN, rate and conduction [15].

## 3. Modern developments in the field

Subsequent cardiac electrophysiological studies greatly advanced our understanding of events underlying pacing, electrical activity and its propagation through specialized conducting tissue into successive atrial, ventricular and conducting regions at the molecular and cellular as well as the systems levels. These studies demonstrated and characterized extensive numbers of novel ion channel, ion transport and receptor protein molecules [21,22]. Many such insights, particularly their translation to roles in normal and arrhythmic activity at the systems level, suggesting novel pharmacological and therapeutic applications, came from monogenically modified murine platforms [23]. Murine and human hearts share dual right- and left-sided circulations, distinct structurally homologous atria and ventricles, and pacing or conducting SAN, AVN and atrioventricular (AV) bundles. They did show differences in size, heart rate, L-type Ca^2+^ current *I*_CaL_ and transient outward K^+^ current contributions (*I*_to_) and consequent APD. Nevertheless, major features of AP depolarization and conduction, transmural conduction velocities, relationships between APDs and ERPs and differences in transmural APD heterogeneities remain conserved [23]. Finally, single cardiomyocyte isolations from these preparations permitted cellular-level experimental studies. In the current theme issue, Salvage *et al*. [24], Remme [25], Terrar [26], Jung *et al*. [27] and He *et al*. [28] review subsequent findings emerging from such genetic platforms; Anderson *et al*. [29] implicate circadian variations in sympathetic actions on pacemaker ion channel gene transcription in diurnal cardiac rate variations in wild-type (WT) murine hearts. Complementary, theoretical, reconstructions then predict the physiological end-effects of the changes observed (Alrabghi *et al*. [30]; Hancox *et al.* [31]).

More recently, genetically modified induced pluripotent stem cell (iPSC) platforms have shown promise, likely as cellular rather than systems models, lacking the anatomically related *in vivo* conducting (Purkinje cell) and contractile (cardiomyocyte) tissue organization involved in initiating and maintaining cardiac arrhythmias. Many available human pluripotent stem cell-derived cardiomyocyte (hiPSC-CM) monolayers show immature embryonic-like as opposed to human adult atrial/ventricular myocardial functional and structural phenotypes, limiting their translational utility [32]. They showed low resting membrane potentials [33], low/absent *I*_K1_ [34], low membrane capacitances [35], immature AP profiles and slow electric impulse propagation velocities [32], and their generation primarily focused on ventricular rather than atrial phenotypes. However, Ahmad *et al*. [36] describe hiPSC-CMs with AP properties and acetylcholine (ACh)-activated *I*_K_ expression characteristic of atrial cells. iPSCs have also been explored as possible models for normal and disease-related changes in ion channel expression, Ca^2+^ homeostatic phenotypes, neurocardiac interactions and cardiac hypertrophic change (see: Chen *et al*. [37]; Zhou *et al*. [38]; Li *et al*. [39] and Langa *et al*. [40], respectively).

Finally, direct human clinical electrophysiological studies continue to generate important scientific and translational insights into cardiac arrhythmic phenomena. Thus, recent electrocardiographic [41,42] and electrical mapping studies [43,44] distinguished potential roles of focal, Purkinje system activity from rotor activity in initiating and maintaining electrophysiologically and pharmacologically distinct polymorphic ventricular tachycardic (VT) or fibrillatory subtypes. These findings have potential implications for the clinical management of post-myocardial infarction sudden cardiac arrest.

This theme issue discusses novel targets and their actions on excitable activity at multiple levels of cardiac functional organization established in this subsequent work as outlined in this introductory review, using standard texts as starting point [45] (figure 1*b*). Thus normal and arrhythmic activity (figure 1*b*(i)) immediately arises from (figure 1*b*(ii)) surface membrane ion channels and their interactions underlying automaticity and pacemaking, and AP excitation, propagation and recovery (§§4 and 5 below). These membrane-level events initiate and are modulated by (iv) cellular-level feed-forward and feedback effects of excitation–contraction coupling and its Ca^2+^-mediated triggering (§6). Both these are modulated by (iii) G-protein-mediated autonomic inputs and the central nervous system circadian rhythms that these may transmit (§7). Of increasing interest are the longer-term regulatory mechanisms related to (v) metabolic feedback (§8) and other upstream target modulators (§9) causing potentially pathological electrophysiological and structural remodelling. All these regulatory events ultimately bear on surface membrane ion channel function in (ii), through which the arrhythmic outcomes emerge. These article sections are keyed to the individual articles in this *Phil. Trans.* theme issue.

## 4. Ion channels contributing to cardiomyocyte surface membrane excitation

Normal cardiac rhythm requires a normal, regular, SAN automaticity. Inward, hyperpolarization-induced cyclic-nucleotide-activated channel (HCN)-mediated *I*_f_ [46] and other ionic currents [47] combine with electrogenic Na^+^/Ca^2+^ exchange (NCX) contributions driven by store Ca^2+^ release [48] (§6). Together these drive a time-dependent membrane potential depolarization from background resting levels to the Ca^2+^ channel threshold. The resulting excitation initiates Na^+^ current and consequent AP excitation at the outer rim of the SAN [49]. Donald & Lakatta [50] review recent discoveries bearing on the coupled-clock system from the cellular level, within the context of a complex cellular SAN organization. This pacing is modulated by adrenergic or cholinergic SAN pacemaker stimulation or inhibition (§7 below). Altered SAN automaticity causing abnormal or altered AP generation can arise from SAN malfunction, SND, or altered background diastolic or resting potentials. Abnormal automaticity can also arise with abnormal AVN or Purkinje tissue pacemaker activity when spontaneous impulses are generated in pathologically partially depolarized fibres, and can even involve normally non-automatic atrial and ventricular muscle. These latter circumstances can cause an automatic, often tachycardic, firing distinct from SAN activity.

**Figure 2.**
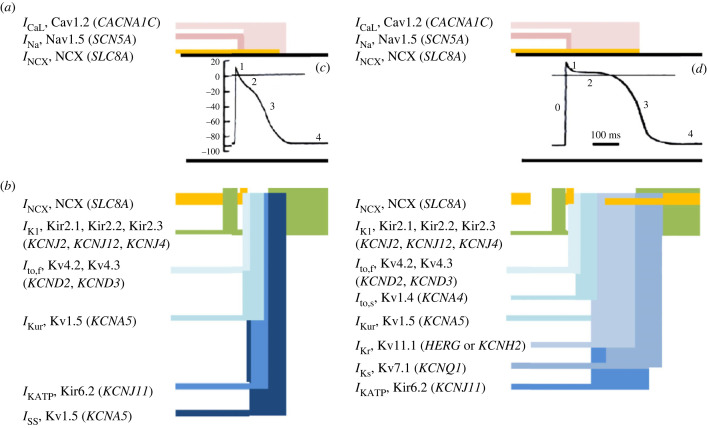
Ion channels underlying atrial and ventricular action potentials. Membrane ion currents, listing their underlying proteins and encoding genes [23], underlying inward depolarizing (*a*) or outward repolarizing currents (*b*) producing phases 0–4 of the atrial (*c*) and ventricular (*d*) action potential. (From fig. 2 of [14]). (Online version in colour.)

The ensuing APs form the functional unit of cardiomyocyte excitable activity. These are driven by a sequence of inward (figure 2*a*) and outward (figure 2*b*) currents mediating successive rapid depolarizing (phase 0), early repolarizing (phase 1), brief atrial (figure 2*c*) and prolonged ventricular (figure 2*d*) plateau (phase 2), late repolarization (phase 3) and electrically diastolic phases (phase 4). Inward *I*_Na_ activation initiates the propagated AP phase as well as the remaining sequence of electrical events. Genetic evidence for loss or gain of *I*_Na_ function correlates with pro-arrhythmic human Brugada (BrS) and long-QT3 syndromes (LQTS3), respectively. Recent findings reviewed here further report feedback actions on *I*_Na_ activation (Salvage *et al.* [24]) and potentially pro-arrhythmic late *I*_NaL_ currents (Liu *et al*. [51]) by further, downstream, excitation–contraction coupling (§5) and metabolic events (§7). All these effects were recapitulated in loss [52,53] or gain of function genetic murine models affecting Nav1.5 [54,55] and RyR2 function [56,57], and metabolic activation [23,58,59]. Furthermore, electrophysiological aberrations and arrhythmic tendency in the BrS and LQTS3 models were similarly accentuated or relieved by flecainide and ameliorated or accentuated by quinidine [53,60], findings with potential translational significance [61,62]. Remme [25] reviews complex Nav1.5 functional and distribution patterns involving particular subcellular cardiomyocyte subdomains, as well as non-canonical non-electrogenic Nav1.5 actions with structural, potentially cardiomyopathic and pro-arrhythmic, effects. Finally, Nav1.5 does occur in other cell types, including various extracardiac tissues. Conversely, cardiomyocytes may express other than Nav1.5 subtypes.

AP conduction involves local circuit currents through connexin channels connecting adjacent cardiomyocytes. Their magnitudes are determined by maximum rates of AP depolarization (d*V*/d*t*)_max_, themselves dependent upon membrane capacitance and cytosolic resistance [63,64]. The resulting AP propagation produces a coherent wave of excitation followed by refractoriness, of wavelength *λ* [23]. This propagates through gap junction connexin and possible ephaptic connections between successive SAN, atrial, AV, Purkinje and endocardial and epicardial ventricular cardiomyocytes [17]. The wavelength *λ* is normally sufficiently long to prevent re-excitation of recovered tissue behind the wave. Abnormal conduction slowing, shortening *λ*, can follow functional reductions in *I*_Na_ or anatomical changes altering tissue electrical resistance or the functional or anatomical conducting pathway (§7; [14,63]). These can also produce heterogeneities in refractoriness and conduction in the conducting circuit. These heterogeneities can vary with time and previous impulse activation, and produce either total and unidirectional conduction block. Finally, at the temporal rather than spatial level, ERPs extend beyond each AP. They can increase with Na^+^ channel inhibition, delaying the point at which a critical proportion of Na^+^ channels have recovered, or with AP prolongation [23].

These changes potentially cause *re-entrant substrate* perpetuating triggering events into sustained arrhythmias [65]. These can involve spatial conduction heterogeneities, exemplified by transmural gradients across the ventricular wall, or temporal heterogeneities with abnormal AP recovery reflecting altered relative timings between AP recovery, refractoriness and repolarization reserve [54,66,67]. Thus, discrepancies between ERP and AP recovery times occur in LQTS. Arrhythmias arising from isolated, decay of or block of impulse conduction can also occur in the absence of re-entrant pathways. Thus, a sino-atrial (SA) conduction block permits escape of a supraventricular or ventricular focus which generates abnormal impulses. Similar phenomena can follow delayed or blocked AV conduction [14].

Different ion channels offer complementary contributions to AP characteristics with differing effects on heart rhythm reflected in turn in different modes of action of particular anti-arrhythmic drugs [68]. Drugs acting on *I*_Na_ alter the AP depolarization phase 0. Of these, Class Ia drugs bind to the Nav1.5 open state with *τ* ≈ 1–10 s dissociation time constants, inhibiting AV conduction and increasing ERPs, additionally increasing APD by a concomitant *I*_K_ block. Class Ib agents bind preferentially to the Nav1.5 inactivated state, from which their more rapid *τ* ≈ 0.1–1.0 s dissociation minimizes their actions through successive cardiac cycles. Class Ic drugs bind to inactivated channels with a slow *τ* > 10 s dissociation giving a use-dependent channel block, slowing AV conduction, but little affecting APD. A new Class Id blocks pro-arrhythmic late Na^+^ current (*I*_NaL_) in LQTS3, and pathological bradycardic and ischaemic conditions, and cardiac failure. Class Id drugs shorten APD and increase refractoriness and repolarization reserve [69].

## 5. Ion channels contributing to cardiomyocyte surface membrane recovery

AP depolarization activates further channels both initiating contraction and restoring the resting membrane potential. The consequent AP waveforms vary with cell type: atrial cells show shorter APs than ventricular cells (figure 2*c,d*) [63,67,70]. Ca^2+^ channel (Cav1.2) activation, localized within the transverse tubules [71], detailed in the next section, contributes to the phase 2 plateau. In certain cardiomyocyte such as SAN and AVN types (see §4), this instead of Nav1.5 initiates excitable activity. Ca^2+^ channel abnormalities can also cause arrhythmic phenotypes [23]. Zeng *et al*. [72] associate variants of pro-arrhythmic J wave syndromes, also found with loss of Nav1.5 function, with loss of Ca^2+^ channel function, *CACNB2b-S143F* and *CACNA1C-G37R*, mutations. AP repolarization ultimately restoring the resting potential is driven by a range of outward K^+^ currents (figure 2*b*) [23,73], for which a wide range of new K^+^ channel subtypes have been described [73–77]. Of these, transient outward Kv4.3 and Kv4.2-mediated *I*_to_ currents drive the early phase 1 AP repolarization terminating phase 0 depolarization. The prominent *I*_to_, together with atrial-specific Kv1.5 (*KCNA5*)-mediated ultra-rapid *I*_Kur_, and the GIRK1- and GIRK4-mediated ACh-sensitive *I*_KACh_, result in the shorter atrial than ventricular APD. Gain of function Kv4.3 and Kv4.2 mutations have been implicated in AF. Alrabghi *et al*. [30] model human atrial cells in computational reconstructions of atrial tissue and intact atria, to replicate reductions in APD, plateau, ERP and consequent *λ*, enhancing AP re-entry and facilitating AF.

In ventricular myocytes, Kv11.1 (HERG or *KCNH2)*-mediated *I*_Kr_ rapidly activates with phase 0 AP depolarization. It then rapidly inactivates over AP phases 0–2 [78,79]. Phase 3 repolarization then re-activates *I*_Kr_, permitting outward phase 3 and early phase 4 currents terminating the plateau. By contrast, Kv7.1 (*KCNQ1*)-mediated *I*_Ks_ activates more slowly over phase 2, becoming a major persistent phase 3 K^+^ conductance. Kir2.1, Kir2.2 and Kir2.3 (*KCNJ2*, *KCNJ12* and *KCNJ4)* mediate inwardly rectifying *I*_K1_. This produces a reduced K^+^ conductance at voltages greater than −20 mV in phases 0–2 while producing outward currents with repolarization to less than −40 mV late in phase 3. It also stabilizes phase 4 diastolic resting potentials. Cardiomyocyte resting potentials are further stabilized by background K_2P_2.1 (*KCNK2*, expressing K_2P_ currents), and the normally small adenosine triphosphate (ATP)-sensitive Kir6.2 (*KCNJ11)* mediating *I*_KATP_. However, the latter can be activated by reduced intracellular ATP levels [80]. Finally, Li *et al*. [81] review effects of further, small-conductance Ca^2+^-activated K^+^ (SK) channels on excitability in both normal and pathological conditions.

*Loss*-of-K^+^ channel function abnormalities are associated with pro-arrhythmic *long*-QT syndromes (LQTS). Computational analysis (Hancox *et al*. [31]) conversely implicates *gain* of K^+^ channel function involving *I*_Kr_, *I*_Ks_ and *I*_K1_ in *short*-QT syndrome (SQTS). The latter also predispose to atrial and ventricular arrhythmias and SCD. Protein expressional and functional changes related to *I*_Ks_ have been closely associated with ventricular arrhythmias. Chen *et al*. [37] reveal a novel role of the ubiquitin-like-modifier leukocyte antigen F-associated transcript 10 (FAT10) in regulating K^+^ channels competing for Kv7.1 ubiquitination. This protects against pro-arrhythmic hypoxia-induced decreases in *I*_Ks_. FAT10 itself protects against myocardial ischaemia. Recent pharmacological targeting of a significant number of these novel K^+^ currents includes new non-selective K^+^ channel inhibitors and drugs directed towards the atrial-specific *I*_Kur_, *I*_Kr_ and *I*_KATP_.

## 6. Ca^2+^ homeostasis and excitation–contraction coupling

**Figure 3.**
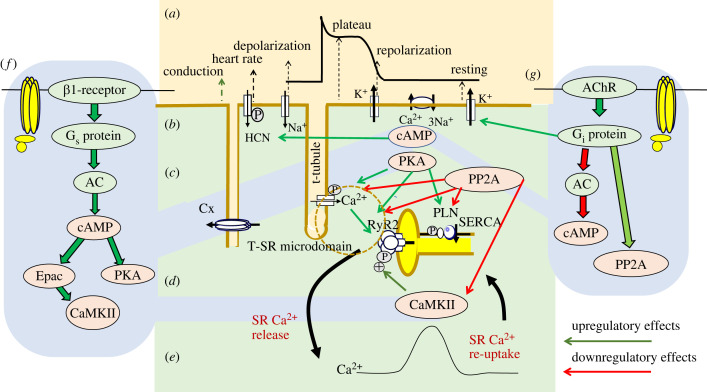
Feed-forward excitation contraction–coupling events and their autonomic modulation. (*a–e*) Relationships between (*a*) surface membrane electrophysiological events resulting in action potential depolarization, plateau, and recovery to resting phase, generated by (*b*) principal underlying ion channels and transporters carrying inward *I*_Na_ and *I*_Ca_, outward *I*_K_, and Na^+^/Ca^2+^ exchange current (NCX). (*c,d*) Consequent homeostatic events involving *I*_Ca_-induced ryanodine receptor (RyR2)-mediated Ca^2+^ release into the transverse tubular–sarcoplasmic reticular (T-SR) Ca^2+^ microdomain (*c*) and SR Ca^2+^ transport (SERCA)-mediated Ca^2+^ re-uptake (*d*) making up the resulting (*e*) cytosolic [Ca^2+^] transient. (*f*) Modulation of Ca^2+^ homeostatic events by sympathetic nervous system activation of stimulatory G-protein G_s_ and its regulatory cellular signalling pathways. Effects of the messengers cyclic 3′,5′-adenosine monophosphate (cAMP), generated by adenylate cyclase (AC), protein kinase A (PKA), exchange protein directly activated by cAMP (Epac) and calmodulin kinase II (CaMKII) on *I*_Ca_, RyR2 and the SERCA regulator phospholamban (PLN), at different stages of the excitation–contraction coupling process. (*g*) Modulation of Ca^2+^ homeostatic events by parasympathetic nervous system activation of inhibitory G-protein G_i_ and regulatory G_α_ and G_βγ_, and protein phosphatase PP2A, cellular signalling pathways. Cx, connexin; HCN, hyperpolarization-induced cyclic-nucleotide-activated channel; AChR, acetylcholine receptor. P denotes phosphorylatable proteins. Upregulatory and downregulatory effects annotated by green and red arrows, respectively. (Online version in colour.)

Figure 3 summarizes the significant progress suggesting reciprocal relationships between membrane excitation and excitation–contraction coupling mechanisms (figure 3*a–d*). Transverse tubular L-type Ca^2+^ current *I*_CaL_ triggering producing the AP phase 2 plateau (figure 3*a,b*) results in extracellular Ca^2+^ entry, causing a local cytosolic [Ca^2+^] elevation in possible Ca^2+^ microdomains formed by membranes bounding the transverse tubule–sarcoplasmic reticular, T-SR, junctions [24,82–84]. This drives *feed-forward* ryanodine receptor (RyR2)-mediated sarcoplasmic reticular (SR) Ca^2+^ release (figure 3*d*). RyRs are additionally regulated by intracellular factors exemplified by the FK506 binding proteins, FKBP12 and FKBP12.6, though their detailed action is debated. Richardson *et al*. [85] report time- and concentration-dependent effects of FKBP12 on previously FKBP12/12.6-depleted RyR2 channels, suggesting negative co-operativity in their FKBP12 binding, potentially significant in regulating RyR-mediated Ca^2+^ signalling. Genetic gain of RyR2 or loss of calsequestrin function is associated with the pro-arrhythmic condition catecholaminergic polymorphic ventricular tachycardia (CPVT) experimentally recapitulated in murine hearts carrying genetically altered RyR2 or calsequestrin-2 [86,87].

The resulting further bulk cytosolic [Ca^2+^] elevation (figure 3*e*) activates troponin, initiating mechanical activity. Ca^2+^ release normally terminates with membrane repolarization. Cytosolic [Ca^2+^] then returns to its resting level through cardiac SR membrane Ca^2+^-ATPase (SERCA2)-mediated Ca^2+^ re-uptake and sequestration by SR calsequestrin, and surface membrane NCX-mediated cytosolic Ca^2+^ extrusion into the extracellular space in exchange for extracellular Na^+^, whose electrogenicity has been implicated in both abnormal rhythm and normal SAN pacing (see §4; Donald & Lakatta [50]) [88]. The cycles of increase followed by restoration of cytosolic Ca^2+^ concentration and therefore of contraction are normally synchronized with membrane events associated with the AP.

**Figure 4.**
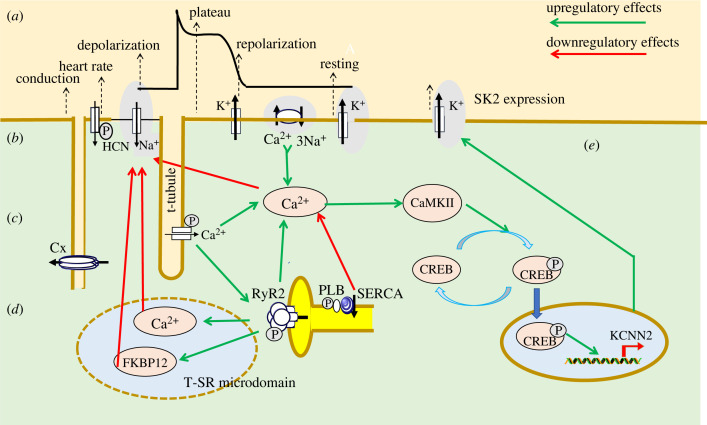
Possible feedback actions of excitation–contraction coupling events on membrane excitation. (*a–d*) Relationships between (*a*) surface membrane electrophysiological events resulting in action potential depolarization, plateau, and recovery generated by (*b*) principal underlying ion channels and transporters and (*c,d*) consequent Ca^2+^ homeostatic events within (*c*), the bulk cytosol, upon *I*_Ca_-induced ryanodine receptor (RyR2)-mediated Ca^2+^ release, SERCA-mediated Ca^2+^ re-uptake, Na^+^/Ca^2+^ exchange and calmodulin kinase II (CaMKII) activity. (*d*) Ca^2+^ homeostatic events within T-SR microdomains, and interactions of released Ca^2+^ and of FK506 binding protein (FKBP12) with Na^+^ and connexin channel activity. (*e*) Longer-term CaMKII actions on transcriptional and translational activity affecting channel activity, exemplified here by cAMP response element-binding protein (CREB)-mediated upregulation of Ca^2+^-activated SK2 K^+^ channels (KCNN2). Cx, connexin; HCN, hyperpolarization-induced cyclic-nucleotide-activated channel. Upregulatory and downregulatory effects annotated respectively by green and red arrows. (Online version in colour.)

Alterations in these excitation–contraction coupling processes potentially exert pro-arrhythmic effects [89,90]. Of *feed-back* effects on their initiating membrane events (figure 4*a*), membrane potential after-depolarization events could elicit triggered activity should their amplitude be sufficient to initiate regenerative Na^+^ or Ca^2+^ channel excitation (figure 4*b*). First, altered *I*_CaL_ could predispose to pro-arrhythmic early after-depolarization (EAD) phenomena late in phase 2 or early in phase 3 of the AP, in turn causing extrasystolic membrane excitation. These events typically occur under bradycardic conditions, when altered balances of inward *I*_Na_ or *I*_Ca_ and outward *I*_K_ prolong the AP. This permits *I*_CaL_ reactivation, which in turn triggers an extrasystolic AP, potentially precipitating *torsades de pointes*. This is particularly likely under acquired or genetic conditions of increased APD exemplified by experimental hypokalaemia or LQTS [54,55].

Secondly, elevated diastolic cytosolic [Ca^2+^] following abnormally increased *I*_CaL_ or RyR2 Ca^2+^ sensitivity can itself trigger propagating waves of spontaneous SR Ca^2+^ release asynchronous to the normal membrane excitation cycles, further elevating cytosolic [Ca^2+^] (figure 4*c*). These can result in delayed after-depolarization (DAD) events that follow full AP repolarization. These are driven by transient inward currents, *I*_ti_, resulting from an electrogenic NCX activity enhanced by the elevated cytosolic [Ca^2+^] produced by the abnormal diastolic SR Ca^2+^ release [23,88,91]. NCX itself may contribute to SAN automaticity through its depolarizing electrogenic effects (see §4; [14,48]). Thirdly, Terrar [26] reviews contributions from further intracellular organelles, including lysosomes and mitochondria, to timing and Ca^2+^ store-based modulation involving further, cADP-ribose, nicotinic acid adenine dinucleotide phosphate (NAADP) and inositol tris-phosphate (IP_3_)-mediated, signalling to intracellular organelles. These further modulations of Ca^2+^ homeostasis may contribute additional arrhythmic mechanisms, often similarly acting through NCX.

Fourthly, elevated cytosolic [Ca^2+^] may also downregulate Na^+^ channel expression and function, compromising AP initiation and/or conduction velocity [92] (figure 4*d*). Salvage *et al*. [24] review this action, likely involving Ca^2+^/calmodulin (Ca^2+^-CaM) and apo-CaM interactions with binding sites on the III–IV linker and the C-terminal domain of Nav1.5 [57]. Such mechanisms appear to operate through a wide range of physiological situations. They could also modify the expression of other ion channels, exemplified by Li *et al*. [81] in the calmodulin kinase II (CaMKII)-mediated modifications in Ca^2+^-activated K^+^ (SK2) channel expression under conditions of cardiac hypertrophy [93], in addition to CaMKII actions in increasing *I*_NaL_ (Liu *et al*. [51]) (figure 4*e*). Finally, Zhou *et al*. [38] report a further possible level of RyR2–Na^+^ channel interaction in iPSCs carrying clinically pro-arrhythmic *RYR2-A1855D*. Their resulting phenotype, with premature spontaneous SR Ca^2+^ transients, Ca^2+^ oscillations and increased APDs, was accentuated by a co-existent *SCN10A-Q1362H* variant by itself not conferring any specific phenotype.

These advances broadened the potential therapeutic anti-arrhythmic options. Ca^2+^ channel blockers can act as non-selective surface membrane Ca^2+^ channel inhibitors. There are also phenylalkylamine and benzothiazepine Cav1.2 and Cav1.3 channel-mediated *I*_CaL_ inhibitors. One RyR2 blocker, flecainide, has found recent use in the monotherapy of CPVT [24,56,94]. Future explorations could target (a) further surface membrane L- and/or T-type Ca^2+^ channels, (b) intracellular RyR-Ca^2+^ channels, (c) SERCA2 activity, (d) ion exchange, particularly Na^+^–Ca^2+^ exchange processes, and (e) phosphorylation levels of cytosolic Ca^2+^-handling proteins, including CaMKII inhibitors, and p21 activated kinase 1 (Pak1) modulators (see §§7 and 9).

## 7. Autonomic G-protein-mediated modulation

The physiological processes of cardiac pacing, ion current activation in AP generation, and the excitation–contraction coupling that initiates myofilament activity are modulated by the cardiac autonomic, sympathetic and parasympathetic innervation (figure 3*f,g*). This releases transmitters and co-transmitters binding to receptors often coupled with guanine nucleotide-binding (G-) proteins. The latter G-protein-coupled receptors (GPCRs) activate regulatory biochemical cascades with complex and multiple inotropic, chronotropic and lusitropic effects upon cardiac function [83]. hiPSC-derived co-culture systems permitting closer examination of neurocardiac interactions are under development. Li *et al*. [39] report one such optimized system replicating many anatomical and pathophysiological features of both the individual and combined cardiomyocyte and innervating components mimicking physiological responses in other mammalian systems.

Sympathetic nervous system terminals are widely distributed through different cardiac regions, where they release noradrenaline (figure 3*f*). Sympathetic activation also triggers adrenal medullary adrenaline release into the circulation. Both transmitters bind to surface membrane β_1_- and β_2_-adrenergic receptors. Of these, the cardiomyocytes express β_1_-adrenergic receptors whose activation triggers widespread actions. Noradrenaline binding activates the stimulatory G-protein G_s_. Its G_α_ subunit binds guanosine triphosphate (GTP) and is released from the receptor and the βγ*-*subunit. The G_α_ subunit then activates the adenylyl cyclase, enhancing cyclic 3′,5′-adenosine monophosphate (cAMP) production, increasing cellular cAMP levels.

First, cAMP combines with, and maintains open, HCN channels, particularly in SAN cells, increasing, pacemaker current *I*_f_ and heart rate. Secondly, cAMP activates protein kinase A (PKA), which exerts widespread strategic phosphorylation actions. The latter include exciting Nav1.5, Kv11.1 and Kv7.1, respectively, mediating rapid inward *I*_Na_ and subsequent outward *I*_Kr_ and *I*_Ks_. PKA also enhances phosphorylation of the C-terminal tail regions of Cav1.2 L-type Ca^2+^ channels, increasing their open probability, increasing both amplitude and duration of the ventricular AP plateau. It also accelerates SAN pacemaker potentials. The consequent increased net Ca^2+^ entry into the cell increases the rate and force of muscle contraction in subsequent beats. PKA-mediated phosphorylation of RyR2 reduces binding of its regulatory ligand FKBP12, which normally stabilizes its closed state. This dissociation increases the Ca^2+^ sensitivity of RyR2, enhancing Ca^2+^-induced Ca^2+^ release. Secondly, PKA-mediated phosphorylation of phospholamban (PLN) relieves its inhibition of SERCA2-mediated re-uptake of previously released cytosolic Ca^2+^, enhancing diastolic SR Ca^2+^ store re-loading. Thirdly, of isoforms of cAMP-dependent exchange proteins directly activated by cAMP (Epac), Epac2 activates CaMKII activity, increasing RyR2-mediated SR Ca^2+^ release [95]. Epac1 activation induces programmes of hypertrophic, morphological and cytoskeletal changes. These accompany increased protein synthesis and induction of cardiac hypertrophic markers mediated by Ca^2+^-dependent calcineurin activation. Tomek & Zaccolo [96] describe cellular compartmentation mechanisms in which such diverse cAMP actions might take place. In addition, different sympathetic responses amongst cardiomyocyte types are exemplified by differing electrophysiological properties and responses to noradrenaline of pulmonary vein compared with left atrial cardiomyocytes. These may contribute to atrial ectopy [97].

Parasympathetic, inhibitory, nerve fibre activity slows heart rates and decreases contractile force. The underlying transmitter, ACh, acts through cardiac muscarinic (M_2_) receptors. ACh–receptor binding activates the coupled G-protein G_i2_. These actions occur in SAN, AVN or atrial myocardium in both the presence and absence, but in ventricular tissue only in the presence, of pre-existing adrenergic challenge. The G_α_ subunit binds GTP and splits off from the receptor and its G_βγ_*-*subunit. G_βγ_ subunits open inward rectifying I_KACh_ or I_KAdo_ channels particularly in supraventricular tissue, by acting on their GIRK1 and GIRK4 components [74,98,99]. This occurs particularly in the SAN but also in atria and ventricles. The dissociated G_iα_ binds to and inhibits adenylate cyclase (AC). This reduces cAMP production in pacemaker cells [100], resulting in their increased *I*_CaL_ and *I*_f_. G_i_ activation may also upregulate protein phosphatase (PP2A) activity. This likely takes place through a reaction sequence involving cell division control protein 42 homologue (Cdc42)/Ras-related C3 botulinum toxin substrate 2 (rac2) and Pak1. PP2A dephosphorylates PKA-phosphorylated proteins at the same serine/threonine phosphorylation sites. It therefore reverses PKA effects on L-type Ca^2+^ channels, RyR2s and the SERCA2a inhibitor PLN. The cardioprotective effects of Pak1 may thus involve increased PP2A activity [101,102] additional to its potentially strategic remodelling actions [103,104] discussed in §9 (He *et al*. [28]; Jung *et al*. [27])*_._* Recent studies have closely examined its actions in increasing SERCA activity [101,102,105].

Finally, adenine nucleotides act as excitatory postganglionic sympathetic co-transmitters on metabotropic P2Y receptors. The resulting adenosine (A_1_) receptor activation activates phosphokinase C (PKC) through phospholipase C-mediated production of diacylglycerol. PKC acts on voltage-gated Na^+^ and K^+^ channels, L-type Ca^2+^ channels and RyR2.

These G-protein-linked systems show significant amplification. Activating a single β-adrenergic receptor activates many G-proteins. Each then activates an enzyme molecule, in turn producing many cAMP molecules. Each activated PKA molecule then phosphorylates several Ca^2+^ channels. Correspondingly, activating one muscarinic receptor produces many G_βγ_ subunits. This opens many GIRK1 channels. Closer characterization of such signalling pathways in iPSC cells is a relatively new area of study. Ahmad *et al*. [36] describe differentiated human iPSCs resembling an atrial phenotype, with the expected electrophysiological and Ca^2+^ signalling properties, and specific transcripts, responsive to adrenergic stimulation, therefore permitting studies of such effects.

Recent results implicate a normal continuous diurnal ion channel remodelling at the level of SAN pacemaking driven by sympathetic, though not parasympathetic, actions coupling central nervous system suprachiasmatic nuclear circadian rhythms to rhythms within the heart itself. These actions were initially attributed to beat-to-beat autonomic transmitter-mediated modulation of specific ion channel activity [106]. A greater adrenal medullary catecholamine release and cardiac catecholamine content might then explain higher awake than asleep resting heart rates [107]. However, recent evidence implicates a periodic transcriptional cardiac remodelling varying ion channel abundances and their consequent ionic current densities in such diurnal heart rate variations. Anderson *et al*. [29] discuss this particularly for the HCN channel, exploring possible mechanisms for these findings. About 44% of the sinus node transcriptome, including many important cardiac ion channels, displays a circadian rhythm [106,108,109]. This non-canonical sympathetic action was reflected in chronic but not acute pharmacological autonomic blockade inhibiting both this circadian rhythm and the related ion channel transcription [106,110]. This could involve cAMP response element action promoting the key clock genes, such as *Per1* and *Per2.18* [111].

The elaboration of adrenergic and cholinergic cardiac actions through fuller understanding of G-protein signalling allows the original Vaughan Williams Class II to be broadened to include G-protein actions in general. These have translated to therapeutic advances in the form of new selective and non-selective adrenergic antagonists, as well as adenosine receptor and cholinergic muscarinic receptor modulators [20]. Possible future potential targets may arise from the numerous (approx. 150) further orphan GPCRs. There are now new non-selective, β-, and selective β_1_-adrenergic receptor inhibitors, muscarinic M_2_ receptor inhibitors and activators, and adenosine A_1_ receptor activators.

## 8. Cardiomyocyte energetics and excitable properties

More recently reported processes affecting longer-term cellular energetics and tissue structure remodelling are also implicated in cardiac arrhythmias. These actions complement the more established acute effects of specific ion channels described above. They are often associated with hypoxic conditions generally [112], hypertrophic or fibrotic change, cardiac failure, ischaemia-reperfusion [113–116] and biochemical conditions including obesity, insulin resistance and type 2 diabetes [117–119]. The resulting oxidative stress and longer-term structural, fibrotic, hypertrophic and inflammatory, changes occur upstream of the membrane-level electrophysiological processes [120–122].

**Figure 5.**
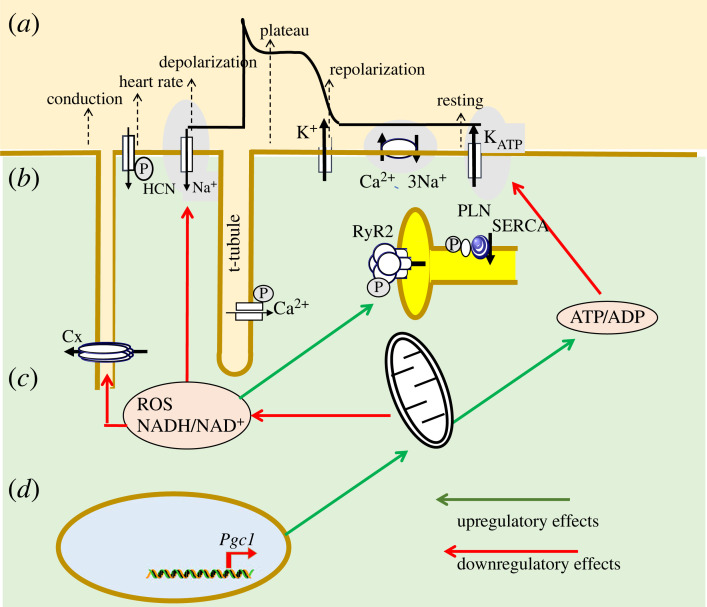
Feedback effects of cardiac energetic changes upon membrane activation and cardiomyocyte Ca^2+^ homeostasis. The presence or absence of arrhythmic phenotype determined at the level of (*a*) surface membrane electrophysiological events resulting in action potential depolarization, plateau, and recovery generated by (*b*) principal underlying ion channels and transporters. However, (*c*) cellular activity involves ATP consumption increasing ADP levels; ATP is normally restored by mitochondrial activity. Excessive energy demand, compromised O_2_ supply or mitochondrial dysfunction increases reactive oxygen species (ROS) production. This inhibits Na^+^ channel function, and oxidizes cardiac ryanodine receptors (RyR2), increasing sarcoplasmic reticular Ca^2+^ leak, increasing cytosolic [Ca^2+^]_i_. The latter itself inhibits *I*_Na_. ROS and the ATP depletion associated with energetic deficiency also open sarcolemmal ATP-sensitive K^+^ channels (sarcKATP) present at high densities in myocyte surface membranes. Finally, cellular levels of reduced or oxidized nicotinamide adenine dinucleotides NADH and NAD^+^, reflecting cell oxidative state, respectively inhibit and enhance Nav1.5 activity. (*d*) Among other transcriptional coactivators, peroxisome proliferator activated receptor γ coactivator-1s (PGC-1), highly expressed in oxidative tissues, regulates mitochondrial mass and function in relation to upstream signals linking cellular energy stores and external demands. PGC1s also regulate expression of key mitochondrial proteins involved in the respiratory chain, mitochondrial fatty acid oxidation and oxidative phosphorylation. ATP/ADP, ATP/ADP ratio; PLN, phospholamban; SERCA, sarcoplasmic reticular Ca^2+^-ATPase; Cx, connexin; HCN, hyperpolarization-induced cyclic-nucleotide-activated channel; upregulatory and downregulatory effects annotated, respectively, by green and red arrows. (Online version in colour.)

Normal cardiomyocyte function in human hearts depends on a number of energy-intensive processes consuming kilogram ATP quantities daily. Approximately 30–40% of this cellular ATP is expended maintaining ionic gradients and efficient Ca^2+^ cycling (figure 5*a,b*). Approximately 90% of the ATP consumption is replenished by the extensive cardiomyocyte mitochondrial network [123–125]. Arrhythmic disorders, particularly AF, have been associated with the metabolic stress associated with metabolic syndrome [126]. Animal models show abnormal mitochondrial structure early following AF induction [127]. Cardiomyocyte mitochondria from human AF patients show increased DNA damage, structural abnormalities and evidence of impaired function [128]. Atrial tissue from chronic AF patients also shows altered transcription of mitochondrial oxidative phosphorylation-related proteins [129]. Decreased mitochondrial complex II/III activity has been reported in permeabilized atrial fibres from patients who developed post-operative AF, corresponding to decreased expression of the gene cluster for mitochondrial oxidative phosphorylation [130]. Finally, right atrial tissue from cardiac surgery patients with an AF history also demonstrated downregulated electron transport chain activity and proton leakage [131].

Mitochondrial dysfunction destabilizes the inner membrane potentials required to drive the electron transport chain, compromising ATP generation. The consequent ATP depletion or rising adenosine diphosphate (ADP) first increases opening probabilities of sarcolemmal K-ATP (sarcKATP) channels [132]. This shortens APDs and consequently the ERPs, predisposing to re-entrant arrhythmia [133,134]. It hyperpolarizes cell membrane potentials, compromising cell excitability and AP propagation [132] (figure 5*c*).

Secondly, excessive energetic demand, compromised vascular oxygen supply or pathological energetic disorders associated with mitochondrial dysfunction also increase reactive oxygen species (ROS) production. The normally occurring low ROS levels modulate activity in a range of signalling molecules or signal themselves. These either transiently alter the activity of proteins, or produce more sustained effects through altering transcription factors and gene expression. ROS influence cardiomyocyte excitability, and atrial and ventricular arrhythmic tendency, effects reduced by allopurinol or ascorbate antioxidant challenge. Increased ROS production could underlie shortened atrial ERPs and initiation of AF with rapid pacing [135,136]. Right atrial appendages of AF patients show increased markers of oxidative stress [131]. Dysregulated ROS production may also reduce cardiac Na^+^ channel expression [137]. In addition, reduced (NADH) or oxidized nicotinamide adenine dinucleotides (NAD^+^), reflecting cell oxidative state, respectively inhibit and enhance Nav1.5 activity, despite normal overall Nav1.5 expression, affecting AP conduction [137]. ROS also reduce connexin-43 (Cx43) trafficking and function [132,138,139] and the consequent cell–cell coupling [140]. Oxidative stress may also influence cardiomyocyte *I*_K_ [141], sarcolemmal K_ATP_ channels [142] and *I*_Ca_.

Thirdly, oxidative stress may also influence Ca^2+^ homeostasis. ROS oxidize RyR2, increasing SR Ca^2+^ leak, increasing cytosolic [Ca^2+^]_i_. It thus altered intracellular Ca^2+^ cycling [116,143,144] in ageing rabbit ventricular myocytes, its effects reversed by a mitochondrial specific ROS scavenger [116]. Oxidative stress also reduces SERCA-mediated Ca^2+^ re-uptake [145]. CaMKII may also be redox-sensitive, with oxidation resulting in kinase activity similar to auto-phosphorylated CaMKII [146]: pharmacological CaMKII inhibition prevented H_2_O_2_-induced ventricular arrhythmias [147]. ROS also oxidize and activate PKA [148]. Finally, ROS may be linked to cardiac fibrosis through fibroblast activation and production of transforming growth factor-β (TGF-β) (§9) [149]. Finally, both CaMKII and ROS could increase *I*_NaL_ (Liu *et al*. [51])

Several transcriptional coactivators regulate mitochondrial mass and function (figure 5*d*) [150]. Of these, the peroxisome proliferator activated receptor (PPAR) γ coactivator-1 (PGC-1) family, including PGC-1α and PGC-1β, is highly expressed in oxidative tissues, including heart, brain, skeletal muscle and kidney. Either PGC-1α or PGC-1β suffices to activate gene regulatory programmes increasing cellular energy production capacity. PGC-1 protein expression increases with a number of upstream signals linking cellular energy stores and external stimuli including cold exposure, fasting and exercise, matching mitochondrial activity to cellular energy requirements. PCG-1s act through numerous nuclear receptor targets including PPARα, PPARβ and oestrogen-related receptor alpha (ERRα). PGC-1α also coactivates nuclear respiratory factor-1 (NRF-1) and -2 (NRF-2) [151]. The latter modulate expression of the nuclear-encoded transcription factor Tfam, essential for replication, maintenance and transcription of mitochondrial DNA [152]. They also regulate expression of other proteins required for mitochondrial function, including respiratory chain subunits [153]. PPARα is also a key regulator of genes involved in mitochondrial fatty acid oxidation. ERRα is an important regulator of mitochondrial energy transduction pathways, including fatty acid oxidation and oxidative phosphorylation [154]. In cardiac cells, PCG-1α interaction with NRF-1, ERRα and PPARα also increases mitochondrial biogenesis [154,155]. Forced PGC-1 expression in cultured cardiomyocytes induced expression of nuclear genes encoding mitochondrial proteins involved in other energy production pathways, including the tricarboxylic acid cycle, and nuclear and mitochondrial genes encoding components of the electron transport chain and oxidative phosphorylation complex [156]. PGC-1 proteins, through these interactions, thus exert multi-level regulation of cellular mitochondrial function and metabolism as a whole.

PCG-1s fall in obesity, insulin resistance, type II diabetes mellitus and ageing in parallel with mitochondrial dysfunction [123,157]. Mice deficient in both *Pgc-1α* and *Pgc-1β* develop a low cardiac output state and conduction system disease, dying before weaning [158]. Ablating either *PCG-1α* or *PGC-1β* produces a milder phenotype, permitting physiological study. *Pgc-1α*^−/−^ hearts have normal baseline contractile function but develop cardiac failure with increased afterload [159]. *Pgc-1β*^−/−^ hearts showed similarly normal baseline features but blunted heart rate responses compared with WT hearts following adrenergic challenge [160]. They also showed an increased arrhythmic propensity. Langendorff-perfused *Pgc-1β*^−/−^ hearts demonstrated APD alternans, and more frequent episodes of VT in response to programmed electrical stimulation [161]. Single-cell studies revealed alterations in the expression of a number of ion channels as well as evidence of spontaneous diastolic Ca^2+^ transients, previously associated with pro-arrhythmic after-depolarizations

Chronic studies of the effects of mitochondrial impairment on the development of pro-arrhythmic phenotypes compared young (12–16 weeks) and aged (older than 52 weeks) *Pgc-1β*^−/−^ mice with aged-matched WT. Chronotropic incompetence in intact animals suggested SND and a paradoxical negative dromotropic response suggested AVN dysfunction, following β_1_-adrenergic challenge [162]. Sharp microelectrode AP recordings in both atria and ventricles of Langendorff-perfused *Pgc-1β*^−/−^ hearts during programmed electrical stimulation demonstrated arrhythmic phenotypes progressing with age. This accompanied reduced (d*V*/d*t*)_max_, prolonged AP latencies, reduced APD, and a consequently reduced AP wavelength (*λ*) correlating with *Pgc-1β^−/−^* arrhythmogenicity [163–165]. These findings could be accounted for by loose patch-clamp demonstrations of reduced *I*_Na_ but not of *I*_K_ in *Pgc-1β*^−/−^ atria and ventricular preparations [58,59]. Finally, the *Pgc-1β*^−/−^ hearts showed accelerated fibrotic change with age (see §9; [165,166]).

## 9. Cardiac remodelling and excitable properties

Remodelling of molecular and physiological processes as well as of cardiac structure can occur over all timescales, and involve any cardiac region(s). There have been recent suggestions implicating non-canonical sympathetic actions in normal diurnal variations in ion channel expression (§7). SAN pacemaking can also be remodelled in disease. Logantha *et al*. [167] report altered SAN ion channel-, Ca^2+^-handling- and fibrosis-related gene expression and implicate these in the SAN dysfunction in a rat pulmonary arterial hypertension model. Investigations of detailed mechanisms are in their infancy. He *et al*. [28] review one line of investigation exploring possible protective signalling actions of PAK1 possibly through altering Cav1.2/Cav1.3 (*I*_CaL_)-mediated Ca^2+^ entry, RyR2-mediated SR Ca^2+^ release and CaMKII-mediated transcriptional regulation of SERCA2a and NCX. Conversely, Jung *et al*. [27] demonstrate that PAK1 deficiency promotes atrial arrhythmogenesis under adrenergic stress conditions, likely through posttranslational and transcriptional modifications of key molecules, including RyR2 and CaMKII, critical to Ca^2+^ homeostasis.

**Figure 6.**
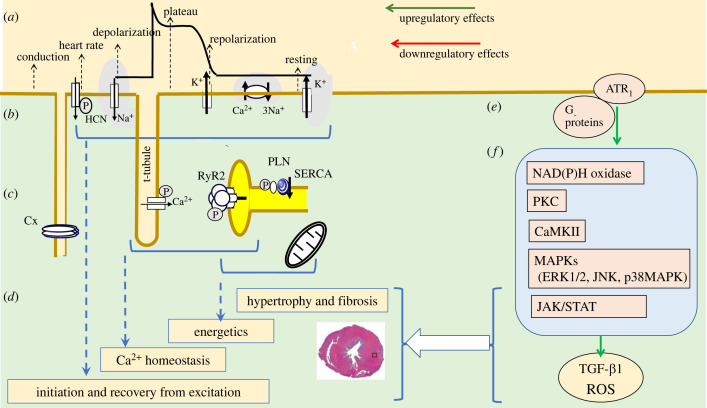
Multicellular-level signalling exemplified by angiotensin II (AngII) action on angiotensin receptor type I, ATR_1_. In addition to effects superimposed on (*a*) surface membrane electrophysiological events generated by (*b*) principal underlying ion channels and transporters and (*c*) the consequent cytosolic Ca^2+^ homeostatic events, such signalling, leads to (*d*) longer-term cardiac hypertrophic and fibrotic remodelling. AngII action involves (*e*) G_αq/11_, G_α12/13_ and G_βγ_ and non-G-protein-related signalling. (*f*) This activates NAD(P)H oxidase, serine/threonine kinases Ca^2+^ calmodulin-dependent protein kinase II (CaMKIII), protein kinase C (PKC) and the mitogen-activated protein kinases (MAPK) extracellular signal-regulated protein kinase 1/2 (ERK1/2), c-Jun NH_2_-terminal kinase (JNK) and p38 mitogen-activated protein kinases (p38MAPK). The latter generate transforming growth factor TGF-β1. It also activates the non-receptor tyrosine kinases Janus kinase/signal transducer and activator of transcription IL (JAK/STAT). These lead to ROS generation, myocardial fibrosis and cardiomyocyte hypertrophy, compromising AP conduction velocity and the integrity of its propagation wavefront. (Online version in colour.)

Longer-term cardiac remodelling involving anatomical, fibrotic and/or hypertrophic change can also occur in cardiac disease processes. The nature of their possible mechanisms are here exemplified by a simplified summary of angiotensin II (AngII) action through its angiotensin receptor type 1 (ATR_1_) (figure 6). Although classically implicated in systemic blood pressure regulation and Na^+^ and H_2_O homeostasis, ATR_1_ activation also stimulates the inflammatory cell recruitment, angiogenesis, cellular proliferation, and accumulation of extracellular matrix (ECM) associated with cardiac hypertrophy and fibrosis [168]. These actions may involve a local cardiac renin–angiotensin system (RAS) thought also to exist in other organs, including blood vessels, brain, kidney, liver and skin. Tissue RASs are functionally autonomous systems of known importance in fibrotic change. They also exert longer-term actions on surface electrophysiological (figure 6*a,b*) and Ca^2+^ homeostatic activity (figure 6*c*), through potential actions of fibrotic and hypertrophic change on AP conduction (figure 6*d*).

ATR_1_s act through both G-protein-, G_α__q/11_, G_α__12/13,_ and G_β__y_, and non-G-protein-related signalling pathways (figure 6*e*), then on multiple, oxidase and kinase signalling pathways (figure 6*f*). These include the serine/threonine kinases CaMKIII and protein kinase C (PKC), and the mitogen-activated protein kinases (MAPK) extracellular signal-regulated protein kinase 1/2 (ERK1/2), c-Jun NH_2_-terminal kinase (JNK) and p38 mitogen-activated protein kinases (p38MAPK). Signalling can also involve receptors, including platelet-derived growth factor (PDGF), epidermal growth factor receptor (EGFR) and insulin receptors, and the non-receptor tyrosine kinases Src, Janus kinase/signal transducer and activator of transcription IL (JAK/STAT) and focal adhesion kinase (FAK) [169].

ATR_1_-mediated NAD(P)H oxidase activation following PKC activation leads to ROS generation, implicated in cardiomyocyte hypertrophy [170,171]. The PKC activation also mediates a galectin-3-dependent fibrosis in HL-1 cells. AngII- or ROS-mediated CaMKII activation, in addition to enhancing phosphorylation of protein targets related to excitation–contraction coupling and cell survival, also did so for transcription factors driving hypertrophic and inflammatory gene expression [172,173]. Activation of the MAPKs, ERK1/2, p38MAPK and JNK, has been implicated in cell growth and hypertrophy [174]. It is also implicated in cardiac fibrosis through increasing gene transcription for procollagen I, procollagen III and fibronectin, and TGF-β, with TGF-β also directly activated by AngII-ATR_1_ binding.

The family of TGFs in turn critically regulates tissue homeostasis and repair, immune and inflammatory responses, ECM deposition, and cell differentiation and growth [175]. TGF-β1, expressed in almost all tissues, is the most prevalent member. TGF-β1 overexpression, acting through Smad, and non-canonically and synergistically through ERK1/2, JNK, and p38MAPK, MAPK signalling, is a key contributor to fibrosis in most tissues [176]. TGF-β1 stimulates myofibroblast differentiation and synthesis of ECM proteins [177] and their preservation, by inhibiting matrix metalloproteinases (MMPs) and inducing synthesis of tissue inhibitor metalloproteinases (TIMPs) [178]. TGF-β1 has been demonstrated to induce fibroblast proliferation, in turn leading to atrial fibrosis [179,180], SND and AF [181]. AngII acts both by itself and in synergy with TGF-β1 to induce fibrosis [168,182]; its fibrogenic effects also have been linked to its activation of TGF-β1 signalling [176,183].

Amongst non-receptor tyrosine kinases, JAK-STAT signalling has been implicated in cardiac hypertrophy and remodelling under conditions of pressure overload and ischaemic pathology [184]. Langa *et al*. [40] discuss emerging data implicating upregulated Notch signalling elements, particularly in hypertrophic (HCM) and dilated cardiomyopathy (DCM), conditions potentially constituting future therapeutic targets in their own right, in variant cTnT-I79N^+/−^ hiPSC-CM cells.

Fibrotic change could be implicated in AF through its action in reducing, and increasing heterogeneities in, AP conduction velocity, and affecting the integrity of AP propagation wavefronts has been implicated in AF. AF also accompanies some Na^+^ channelopathies [185,186]. Therapeutic exploration within this area has thus far targeted remodelling processes rather than their consequent electrophysiological properties. This is exemplified by now-available angiotensin-converting enzyme and angiotensin receptor blockers, aldosterone receptor antagonists, 3-hydroxy-3-methyl-glutaryl-CoA reductase inhibitors (statins), and *n*-3 (ω−3) polyunsaturated fatty acids [121]. Nevertheless anti- arrhythmic drugs in this class may be possible [103,104]. Thus, the key cardiomyocyte regulator of ion channel activity, Ca^2+^ homeostasis and cardiac contractility [101,102,105], PAK1 may offer cardioprotective actions through inhibiting maladaptive, pro-arrhythmic, hypertrophic remodelling and progression in cardiac failure [187,188], actions of possible therapeutic utility (He *et al*. [28]; see also §§6 and 7).

## 10. Cycles of physiological discovery and their clinical translation

The developments outlined here extend Weidmann's initial key electrophysiological studies and Vaughan Williams's classification of cardiac drugs and physiological and therapeutic targets, and have resulted in the development of novel, therapeutic classification schemes. The updating by a Working Group of the European Society of Cardiology [189] provided a more complete, flexible pathophysiological framework predicting pro-arrhythmic circumstances, often termed the Sicilian Gambit [190–192]. However, this did not seek or find extensive use as a formal classification scheme. A more recent reclassification of pharmacological targets and anti-arrhythmic agents [68] related the more recently characterized ion channels, transporters, receptors, intracellular Ca^2+^- handling and cell-signalling molecules to their physiological, and potential and actual therapeutic actions. These were organized by strategic aspects of cardiac electrophysiological function paralleling the coverage in this *Phil. Trans. B* theme issue (figure 1*a*(ii),*b*). In so doing it was possible also to classify both existing and potential cardiac drugs and currently acceptable and potential sites of drug action.

This classification also sought to facilitate future developments of investigational new anti-arrhythmic drugs. It expanded and updated established Singh–Vaughan Williams classes, in particular introducing target classes encompassing the longer-term processes in §§8 and 9. It added to Class I *I*_NaL_ components with implications for long QT syndrome type 3 (LQTS3). A broadened Class II more fully dealt with G-protein signalling, and an expanded Class III incorporated subsequently discovered K^+^ channel subtypes. A much increased Class IV encompassed recent findings on Ca^2+^ homeostasis and excitation–contraction coupling. New classes recognized SAN automaticity (Class 0), and mechanically sensitive (Class V) and gap junction channels (Class VI), and longer-term energetic changes and structural remodelling (Class VII). The revised scheme thus provided a simple working model for cardiomyocyte function in which arrhythmia followed abnormal cardiac electrophysiological activation, linking particular therapies with then-known mechanistic targets (referenced in [68]).

The physiological sciences have long worked in a succession of cycles involving mutually reinforcing interactions between laboratory and clinic. Identification of a clinical problem, particularly its aetiology, epidemiology, diagnosis, and natural history, or of novel physiological phenomena, prompts development of experimental models for the related disease process. These could augment mechanistic and clinically translatable understanding currently incomplete even for common and important arrhythmic conditions such as AF (Hu *et al*. [193]). The resulting physiological insights would prompt clinical tests and explorations for management and treatment. In turn, feedback of the outcomes of these continues the iterative cycles of experimental and clinical testing, activities currently termed translational medicine, for which some current efforts have been recently summarized (see supplementary file in [68]). The particular cycle of efforts represented in this present issue might then prompt further attempts at usefully determining physiological targets for investigational new drugs and other interventions directed at cardiac arrhythmic disease.

## Data Availability

This article has no additional data.
